# Transcriptome-wide *N* ^6^-Methyladenosine Methylome Profiling Reveals m^6^A Regulation of Skeletal Myoblast Differentiation in Cattle (*Bos taurus*)

**DOI:** 10.3389/fcell.2021.785380

**Published:** 2021-12-06

**Authors:** Xinran Yang, Jianfang Wang, Xinhao Ma, Jiawei Du, Chugang Mei, Linsen Zan

**Affiliations:** ^1^ College of Animal Science and Technology, Northwest A&F University, Yangling, China; ^2^ National Beef Cattle Improvement Center, Northwest A&F University, Yangling, China

**Keywords:** *N* ^6^-methyladenosine, cattle, myoblast differentiation, m^6^A-seq, differentially methylated genes

## Abstract

*N* ^*6*^-methyladenosine (m^6^A) is the most prevalent methylation modification of eukaryotic mRNA, and it plays an important role in regulating gene expression. Previous studies have found that m6A methylation plays a role in mammalian skeletal muscle development. However, the effect of m^6^A on bovine skeletal myogenesis are still unclear. Here, we selected proliferating myoblasts (GM) and differentiated myotubes (on the 4th day of differentiation, DM) for m^6^A-seq and RNA-seq to explore the m^6^A methylation modification pattern during bovine skeletal myogenesis. m^6^A-seq analysis revealed that m^6^A methylation was an abundant modification of the mRNA in bovine myoblasts and myotubes. We scanned 5,691–8,094 m^6^A-modified transcripts, including 1,437 differentially methylated genes (DMGs). GO and KEGG analyses revealed that DMGs were primarily involved in transcriptional regulation and RNA metabolism, as well as insulin resistance and metabolic pathways related to muscle development. The combined analysis further identified 268 genes that had significant changes at both m^6^A and mRNA levels, suggesting that m^6^A modification may regulate myoblast differentiation by mediating the expression of these genes. Furthermore, we experimentally confirmed four genes related to myogenesis, including MYOZ2, TWIST1, KLF5 and MYOD1, with differential changes in both m^6^A and mRNA levels during bovine myoblast differentiation, indicating that they can be potential candidate targets for m^6^A regulation of skeletal myogenesis. Our results may provide new insight into molecular genetics and breeding of beef cattle, and provide a reference for investigating the mechanism of m^6^A regulating skeletal muscle development.

## Introduction

Skeletal muscle is an important factor that regulates livestock muscle quality and maintains metabolic homeostasis (Picard et al., 2010). The growth and development of skeletal muscle are extremely complex biological processes, which successively include directional differentiation of progenitor cells, myoblast proliferation, differentiation and fusion of myocytes, and, finally, formation of multinucleated muscle fibers with contractile function (Braun and Gautel, 2011). Besides a series of specific transcription factors, epigenetic modifications such as DNA methylation and histone methylation also play an important role in skeletal myogenesis (McKinnell et al., 2008; Yang et al., 2021). Nonetheless, molecular selection breeding in beef cattle mostly focused on the exploration of some key genes, and rarely improved the breeding process from the perspective of RNA.

More than 150 kinds of chemical RNA modifications have been identified, and RNA methylation accounts for more than 60% of all modifications (Cantara et al., 2011). Among these types of modification, *N* ^6^-methyladenosine (m^6^A) is considered the most prevalent internal mRNA modification in eukaryotes (Wei et al., 1975, 1976; Schibler et al., 1977; Zhong et al., 2008; Jia et al., 2013; Schwartz et al., 2013; Luo et al., 2014; Meyer and Jaffrey, 2014; Haussmann et al., 2016; Shen et al., 2016). m^6^A is a dynamic and reversible posttranscriptional methylation modification (Fu et al., 2014; Meyer and Jaffrey, 2014; Zhao et al., 2017), that is catalyzed by m^6^A writer proteins (methyltransferase complexes composed of methyltransferase-like 3 (METTL3), METTL14 and Wilms tumour 1-associated protein (WTAP)) and is demethylated by m^6^A eraser proteins [fat mass and obesity-associated protein (FTO) and AlkB homolog H5 (ALKBH5)] (Jia et al., 2011; Zheng et al., 2013; Liu et al., 2014; Ping et al., 2014; Shi et al., 2019). The m^6^A modification is functionally interpreted by m^6^A “reader” proteins, such as the widely studied YTH-domain family proteins (Zhang et al., 2010; Wang et al., 2014). m^6^A modification plays a variety of roles in mRNA metabolism, including mRNA translation efficiency, stability, splicing, and nuclear export (Dominissini et al., 2012; Fu et al., 2014; Wang et al., 2014; Zhao et al., 2014; Alarcon et al., 2015; Meyer et al., 2015; Wang et al., 2015; Shi et al., 2019). Accumulating evidence suggests that m^6^A affects different developmental and biological processes, such as multiple cancer processes, mESC differentiation, antitumor immunity, embryonic and postembryonic development, cell rhythms, cell fate determination, and adipogenesis (Fustin et al., 2013; Batista et al., 2014; Zhao et al., 2014; Lin et al., 2016; Bertero et al., 2018; Yang et al., 2018; Han et al., 2019; Song et al., 2019; Yao et al., 2019; Wang et al., 2020).

Recent advances in the field have shown that m^6^A modification plays an important role in processes related to muscle growth and development, such as myocyte stem maintenance, myocyte proliferation, cell differentiation and myocardial function (Kudou et al., 2017; Wang et al., 2017; Dorn et al., 2019; Mathiyalagan et al., 2019; Gheller et al., 2020; Lin et al., 2020; Zhang et al., 2020). In primary mouse myoblasts and C2C12 cells, m^6^A is essential for skeletal muscle differentiation (Kudou et al., 2017; Li et al., 2021) and regulates the transitions of muscle stem cells/myoblasts (Gheller et al., 2020). In farm animals, using m^6^A-specific methylated RNA immunoprecipitation coupled with next-generation sequencing (m^6^A-seq), Tao revealed the m^6^A modification maps in porcine adipose and muscle tissue (Tao et al., 2017), Cheng found the m^6^A modification pattern in chicken fat deposition (Cheng et al., 2021), and Xu uncovered the m^6^A regulation in goose embryonic muscle development (Xu et al., 2021). These studies indicated that m^6^A modification plays a potentially important role in adipogenesis and myogenesis of animals. However, the studies on m^6^A in livestock and poultry mainly focus on the tissue development level, while the transcriptome-wide m^6^A methylome maps in cellular level have been seldom reported. Moreover, m^6^A modification in bovine skeletal muscle development and myogenic differentiation has not been reported.

The present study was aimed to uncover the m^6^A modification pattern in bovine skeletal myoblast differentiation and explore the potential function of m^6^A modification during myoblast differentiation. We used bovine skeletal myoblasts as a research target to investigate the abundance, function and mechanism of m^6^A modification in the process of myogenic differentiation. Thus, we performed m^6^A-seq and RNA-seq in pre-differentiation (GM, myoblasts) and post-differentiation (DM, myotubes) cells. Our results indicate that m^6^A modifications were highly enriched in mRNA, especially in the 3′UTR and CDS regions, and were likely to participate in the regulation of myogenic differentiation. Finally, we identified, screened and verified four skeletal muscle development-related genes (MYOZ2, TWIST1, KLF5 and MYOD1), which showed significant differences in both m^6^A methylation and mRNA expression. Our study first revealed the mRNA m^6^A modification map during bovine skeletal myogenic differentiation *in vitro*, which could contribute to further understand the roles of m^6^A in bovine skeletal muscle development.

## Materials and Methods

### Ethical Statement

The animal experiments of this study were conducted in the light of the protocol of the Experimental Animal Management Committee of Northwest A&F University (Protocol NWAFAC1120), and in accordance with the Regulations on Administration of Animals Used as Subjects of Experiments issued by the State Council of China in 2017.

### Isolation and Culture of Bovine Myoblasts

Primary myoblasts were isolated from the longissimus dorsi of a newborn Qinchuan beef cattle according to the method previously reported in our laboratory (Wang et al., 2018). The isolated myoblasts were cultured to 80% confluence in growth medium, and then, myogenic differentiation was induced with differentiation medium. The culture conditions were a humidified incubator (Thermo Fisher Scientific, MA, USA) containing 5% carbon dioxide at 37°C. The myoblast growth medium was composed of Dulbecco’s modified Eagle’s medium: Nutrient Mixture F-12 (DMEM/F12; HyClone, USA), 20% fetal bovine serum (FBS; GIBCO, USA) and 1% penicillin/streptomycin. The myoblast differentiation medium consisted of DMEM/F12 containing 2% horse serum (HS; GIBCO, USA) and 1% penicillin/streptomycin. The medium was changed every 2 days.

### Immunofluorescence

Cultured myoblasts and myotubes were washed briefly with PBS and fixed with PBS containing 4% paraformaldehyde for 20 min at room temperature, and then permeabilized with PBS containing 0.2% Triton X-100 (Solarbio, Beijing, China) for 10 min. The cells were subsequently washed with PBS 3 times. The cells were blocked with 10% donkey serum, 1% BSA and 0.3 M glycine in PBS at room temperature for 30 min. The primary antibodies were diluted to different concentrations in blocking buffer according to the protocols, and then, the cells were incubated overnight at 4°C. After washing three times with PBS, the cells were incubated with fluorescent dye-conjugated secondary antibodies diluted in blocking buffer for 1 h at room temperature, and this step was performed in the dark. The cells were washed 3 times with PBS, stained with 0.1% DAPI (Sigma-Aldrich, USA) for 15 min and then visualized under a fluorescence microscope (Olympus IX71, Japan). The primary antibodies used were anti-PAX7 (1:200, ab187339, Abcam) and anti-MyoD1 (1:200, ab16148, Abcam). The secondary antibodies used were Alexa Fluor 555-conjugated donkey anti-rabbit IgG (1:1,000, ab150074, Abcam) and Alexa Fluor 488-conjugated goat anti-mouse IgG (1:1,000, ab150113, Abcam).

### RNA Isolation and Fragmentation

Proliferating myoblasts (named GM; 80% confluence, cultured in GM) and differentiated myotubes (named DM; cultured in DM for 4 days) were harvested, and total RNA was extracted using RNAiso reagent (TaKaRa, Dalian, China). Then the RNA samples were sent to LC-BIO Bio-tech ltd. (Hangzhou, China) for RNA sequencing and m^6^A sequencing. The quality and quantity of the total RNA were analyzed by Bioanalyzer 2100 and RNA 6000 Nano LabChip Kit (Agilent, CA, USA) with RIN >7.0. Poly (A) mRNA was isolated from total RNA over 200 ug using poly-T oligo attached magnetic beads (Invitrogen, Massachusetts, USA). Following purification, the poly(A) mRNA fractions are fragmented into about 100-nt-long oligonucleotides by divalent cations at high temperatures.

### m^6^A Immunoprecipitation, Library Construction and Sequencing

The cleaved RNA fragments were incubated with m^6^A-specific antibody (202003, Synaptic Systems, Germany) in IP buffer (50 mM Tris-HCl, 750 mM NaCl and 0.5% Igepal CA-630) supplemented with BSA (0.5 μg/μl) at 4°C for 2 h. Then the mixture was incubated with protein-A beads and eluted with elution buffer (1 × IP buffer and 6.7 mM m^6^A). The eluted RNA was precipitated with 75% ethanol. According to the strand-specific library prepared by dUTP method, the eluted fragments containing m^6^A (IP) and the untreated input control fragments were converted to final cDNA library. The average insert size for the paired terminal libraries was ∼100 ± 50 bp. And then we performed the paired-end 2 × 150 bp sequencing on an Illumina Novaseq™ 6000 platform at the LC-BIO Bio-tech ltd. (Hangzhou, China) following the vendor’s recommended protocol.

### RNA-Seq Data Analysis

*RNA-seq alignment.* Trimmomatic (Bolger et al., 2014) were used to remove the contained adaptor contamination and low-quality bases, Then, we used the fastQC software to verify the sequence quality of each sample. We mapped valid reads to the reference genome of *Bos taurus* (ARS-UCD1.2) published on Ensembl website using HISAT2 (Kim et al., 2015). Then StringTie (Pertea et al., 2015) was accessed to quantify the expression level of all genes and transcripts by calculating FPKM [total exon fragments/mapped reads (millions) × exon length (kB)]. And reads were counted by featureCounts software (Liao et al., 2014).

Differential analysis. Differential expression analysis was performed using edgeR (https://bioconductor.org/packages/edgeR), a Bioconductor package in R, and marked with significant parameter. The differentially expressed genes (DEGs) were selected with Fold Change (FC) > 1.5 or FC < −1.5 and *p* value <0.05. The R package ggplot2 was used to generate differential volcano maps.

GO and KEGG analysis*.* Gene Ontology (GO) classification and enrichment analysis was performed by g:Profiler online tool (Raudvere et al., 2019). Kyoto Encyclopedia of Genes and Genomes (KEGG) was analyzed by KOBAS online website (Bu et al., 2021). Bonferroni and Hochberg corrected *p* value <0.05 in individual genes was considered to be statistically significant.

### m^6^A-Seq Data Analysis

m^6^A-seq alignment and peak calling. After obtaining raw sequence data, we first conduct quality control for raw reads by fastQC and trim low-quality reads and adapter sequences by Trimmomatic (Bolger et al., 2014). Trimmed reads were called clean reads, and were aligned to the *Bos taurus* reference genome (ARS-UCD1.2) using HISAT2 software (Kim et al., 2015). The Uniquely aligned sequences were extracted by Sambamba (Tarasov et al., 2015) and only the uniquely mapped and non-duplicated alignments were further analyzed. MACS2 (Zhang et al., 2008) was used to identify the m^6^A-modification peaks of each sample with the default parameters, which identifies m^6^A peaks with bed or bam format that can be adapted for visualization on the Integrative Genomics Viewer (IGV) software (http://www.igv.org/). *De novo* and known motif were found using MEME (Bailey et al., 2009) and HOMER (Heinz et al., 2010), and Perl scripts in house were used to locate the motif with respect to peak summit. Meanwhile, the input RNA sequencing (RNA-seq) data were used as the background when calling peaks.

Overall analysis of m^6^A data*.* Deeptools (Ramirez et al., 2016) was used to analyze the correlation between samples and reads enrichment signal. ChIPseeker (Yu et al., 2015) were used to annotate the peaks. Bedtools (Quinlan and Hall, 2010) was used to count the peak number of each bin, and the counts were employed to plot the patterns by R. Next, Guitar (Cui et al., 2016) was used to examine the distribution pattern of the m^6^A peaks throughout different regions of the transcripts, the mRNA transcripts were divided into five non-overlapping segments: the 5′UTR, start codon (100 nucleotides centered on the start codon), CDS, stop codon (100 nucleotides centered on the stop codon), and 3′UTR. Each area was separated into 20 bins. Circos analysis was performed using the OmicStudio tools at https://www.omicstudio.cn/tool/.

Analysis of differential peaks between GM and DM*.* Correlation Heatmap was generated by DiffBind (Stark and Brown, 2011). All m^6^A peaks in growth and differentiation conditions for myoblasts were identified by the exomePeak (Meng et al., 2014). For differential analysis, we retrieved all peaks with >1.5-fold differences for downstream analysis. The analysis of GO and KEGG was the same as RNA-seq. Finally, we analyzed the correlation between m^6^A abundance and mRNA level during myoblast differentiation using R packages. The scatter plots of correlation were generated using ggplot2 (v3.3.5), the Pearson correlation coefficient was calculated using ggpubr (v0.4.0), and the fitting equation was added to the plots by ggpmisc (v0.4.2).

### cDNA Synthesis and Real-Time Quantitative PCR

The PrimeScript RT reagent kit (TaKaRa) was used to synthesize cDNA. The residual genomic DNA was removed at 42°C for 2 min, and then, the reverse transcription reaction was conducted at 37°C for 15 min and then at 85°C for 5 s. Real-time quantitative PCR (RT-qPCR) was performed using the TB Green Premix Ex Taq II Kit (TaKaRa) and a CFX Connect Real-Time PCR Detection System (BIO-RAD, CA, USA). Bovine *GAPDH* was used as the internal reference to standardize the data. Each sample analyzed by RT-qPCR was subjected to at least three biological repeats. Relative mRNA expression was calculated and analyzed using the 2^−ΔΔCt^ method (Livak and Schmittgen, 2001). All of the primers used in the RT-qPCR are listed in Supplementary Table S1.

### m^6^A-IP-qPCR

m^6^A immunoprecipitation assays were performed as previously described (Dominissini et al., 2012). In brief, 48 h after transfection, RNA from the cells was chemically digested into 200-nt fragments, and more than 200 μg of total RNA was subjected to immunoprecipitation with affinity-purified m^6^A-specific antibody (202003, Synaptic Systems, Germany). The RNA fragments that bound to m^6^A were separated by TRIzol reagent. Following ethanol precipitation, the input RNA and eluted m^6^A RNA were reverse transcribed by random hexamers, and then, the enriched sequences were detected by RT-qPCR. The ΔΔCt between the 10% input and the immunoprecipitated RNA was determined, and the relative enrichment was calculated as 2^−ΔΔCt^. The primers used to amplify the m^6^A peak region are listed in Supplementary Table S1.

### Statistical Analysis

All data were presented with the means ± standard deviation (SD) of at least three biological repeat samples. Student’s *t*-test (between two groups) or ANOVA (among multiple groups) were used to compare significance of the mean values. Differences were considered to be very significant or significant at *p* < 0.01 or *p* < 0.05, respectively. The results were analyzed using GraphPad Prism 7.00 (CA, USA) software and images were generated.

## Results

### Identification of Bovine Skeletal Myoblasts

**FIGURE 1:**
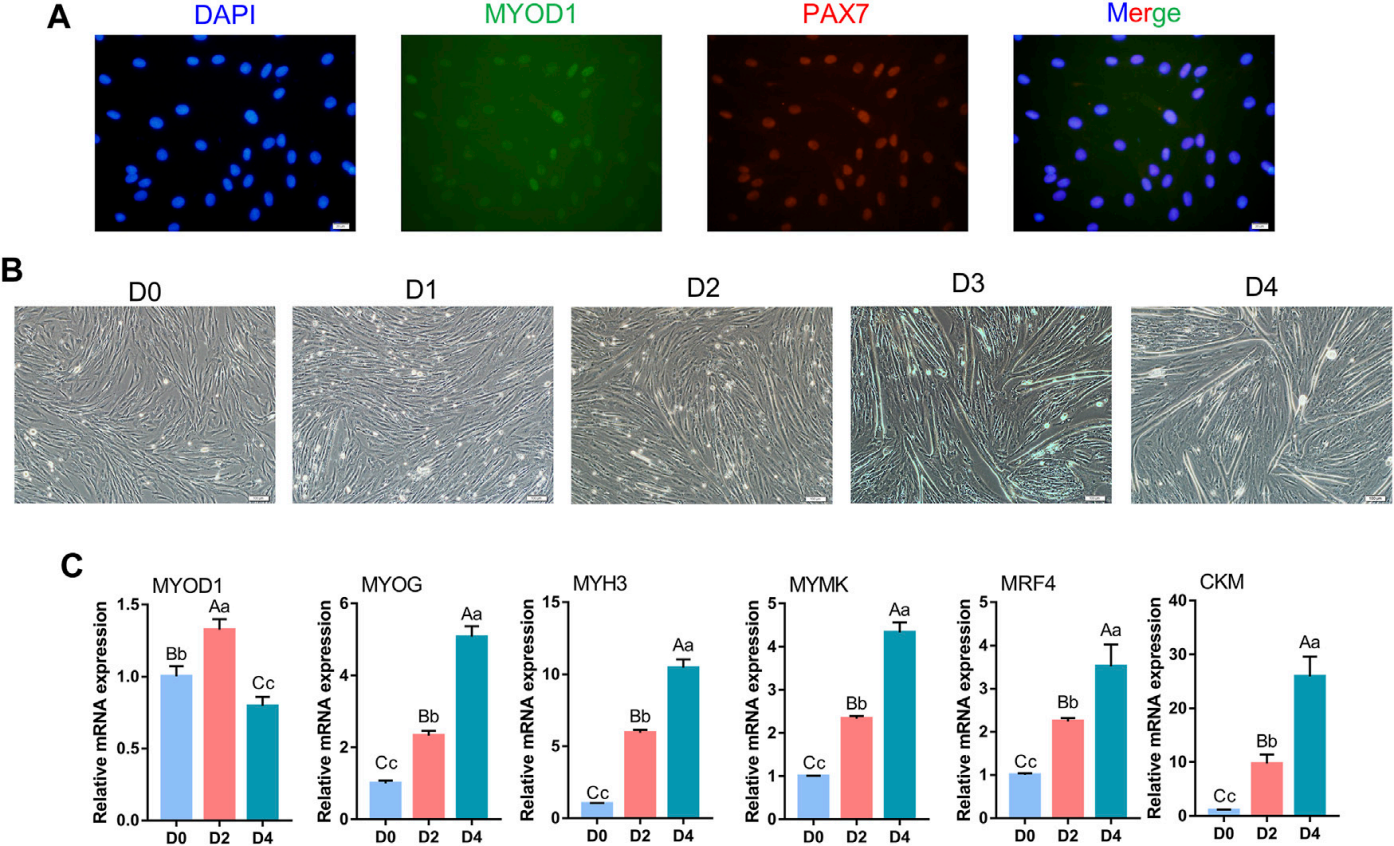
Identification of bovine skeletal myoblasts. **(A)** Identification of myoblasts based on PAX7 and MYOD1 expression in growth medium (GM) (scale bar: 20 μm). **(B)** Observation of myotube formation on days 0, 1, 2, 3 and 4 of culture in differentiation medium (DM) after the induced myogenic differentiation (scale bar: 100 μm). **(C)** Relative mRNA expression of specific myogenic genes (*MYOD1*, *MYOG*, *MYH3*, *MYMK*, *MRF4* and *CKM*) during bovine skeletal myoblast differentiation. The results were normalized to the *GAPDH* levels and were presented as the means ± SD. Different capital letters indicate very significant differences (*p* < 0.01), different lowercase letters indicate significant differences (*p* < 0.05), and the same letters indicate no significant differences (*p* > 0.05).

To verify whether the cells isolated from bovine longissimus dorsi muscles could undergo myogenic differentiation, we seeded the isolated cells in culture dishes, grew them to 80–90% confluence, and passaged them to a 6-well plate for further culture. After 48 h in the growth medium, immunofluorescence showed that PAX7 and MYOD1 were simultaneously expressed, while the expression of MYOD1 was relatively low (Figure 1A). Therefore, we preliminarily identified the isolated cells as myoblasts. The myoblasts were cultured in growth medium to 90% confluence, and myogenic differentiation was induced with differentiation medium. Microscopic observation revealed that the myotubes formed by myoblast fusion gradually increased and became longer as the number of days of differentiation increased (Figure 1B). We evaluated the differentiation status of the myoblasts by detecting the mRNA levels of *MYOD1*, *MYOG*, *MYH3* (myosin heavy chain 3), *MYMK* (myomaker, myoblast fusion factor), *MRF4* (myogenic regulatory factor 4) and *CKM* (creatine kinase), which are widely recognized marker genes of differentiated myoblasts and fused myotubes (Bentzinger et al., 2012; Millay et al., 2013). Notably, the levels of *MYOG*, *MYH3*, *MYMK*, *MRF4* and *CKM* gradually increased during myogenic differentiation, and the trends in the expression of these pivotal genes were consistent with the differentiation stage (Figure 1C). Alternatively, the levels of *MYOD1* peaked on D2 (Figure 1C), which was consistent with previous studies showed that MYOD1 plays a vital role in the proliferation and early differentiation of myoblasts (Weintraub et al., 1991; Weintraub, 1993). These results indicated that the isolated bovine skeletal myoblasts in this study were capable of myogenic differentiation and could serve as a model for our follow-up study.

### Overview of Samples and *N* ^6^-Methyladenosine Methylation of mRNA in Bovine Sketetal Myoblasts and Myotubes

**FIGURE 2:**
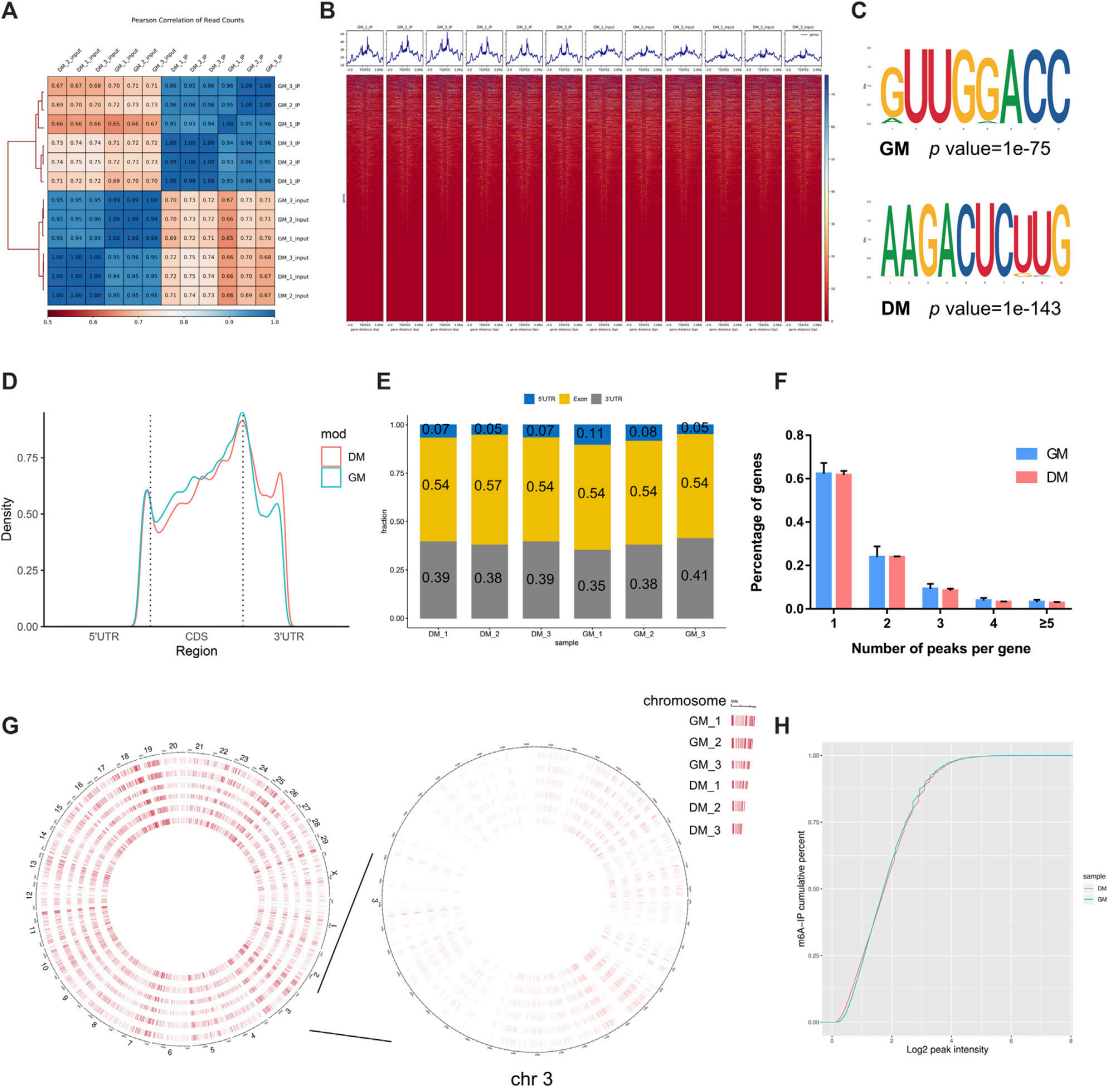
Overview of samples and m^6^A methylation of mRNAs in bovine skeletal myoblasts and myotubes. **(A)** Pearson correlation analysis showing the correlation between the groups, represented as values in modules. **(B)** Distribution of peaks on gene functional elements in all groups. **(C)** Sequence motifs identified within m^6^A peaks. **(D)** Metagene profiles of m^6^A peaks distribution across the 5′ UTR, CDS, and 3′ UTR in GM and DM. **(E)** Percentage of m^6^A peak in 5′ UTR, CDS, and 3′ UTR of mRNA. **(F)** Percentage of m^6^A-methylated genes with different number of m^6^A peaks. **(G)** Circos plot showing the global distribution of m^6^A, in cow genome (left) and chromosome 3 (right) of GM and DM. **(H)** Cumulative curves of m^6^A abundance in GM and DM.

To investigate the role of m^6^A in myoblast differentiation, mRNA was extracted from pre-differentiation (GM, myoblasts, D0) and post-differentiation (DM, myotubes, D4) cells for m^6^A-seq and RNA-seq. Pearson correlation analysis showed that there was a strong correlation between the three biological repeat samples in GM and DM groups, respectively (Figure 2A). RNA-seq and m^6^A-seq produced 46785592–66324558 clean reads in Input and IP groups, of which more than 95% were mapped to reference genome of *Bos taurus* (Table 1). After eliminating low-quality reads, more than 92% of unique mapped reads were obtained in each group of clean reads (Table 1). These data show that high-throughput sequencing in this study has been carried out successfully. Furthermore, we identified 23287–23912 total transcripts in GM and DM groups (Table 2, Supplementary Table S2). Then the methylated mRNA was mapped to the transcriptome. 6,042–8,094 m^6^A-modified transcripts were identified in GM groups and 5,691–7,361 m^6^A-modified transcripts were found in DM groups. There were 9,246–13596 and 9,128–11861 m^6^A peaks in the two groups, respectively (Table 2, Supplementary Table S3). Therefore, we found that there were ∼1.60 m^6^A peaks per m^6^A transcript in bovine skeletal myoblasts and myotubes, which were similar to those detected in human HepG2 (∼1.7 peaks per gene) (Dominissini et al., 2012), pig muscle (∼1.7 peaks per m^6^A transcript) (Jiang et al., 2019), pig adipose tissue (∼1.3 peaks per gene) (Tao et al., 2017) and chicken fat (∼1.5 peaks per m^6^A transcript) (Cheng et al., 2021). The results of the three biological replicates were similar, which also showed the accuracy of m^6^A-seq results and the similarity between duplicate samples. Summary plots and heatmaps were generated by deepTools using normalized read coverages from m^6^A-seq in bovine skeletal myoblasts (Figure 2B). The Summary plots on top of the heatmap (Figure 2B, top panel) indicate that the enrichment intensity between and near TSS (transcription start site) and TES (transcription end site) in the genome region of the immunoprecipitation (IP) group is higher than that in the Input group, and peaks signal is most strongly enriched around the start and end of genes which is also visible in the heatmaps. Clustering of significantly enriched sequences found m^6^A consensus motif RRACH was highly enriched in GM and DM (Figure 2C), which was consistent with the general pattern of mammals.

**TABLE 1:** Summary of sequencing data and read-alignment statistics from m^6^A-seq in bovine skeletal myoblasts and myotubes.

Sample	Clean reads	Mapped reads	Unique mapped reads
GM_1_IP	46,785,592	44,867,383 (95.90%)	43,481,742 (92.94%)
GM_2_IP	59,442,772	56,964,008 (95.83%)	55,031,796 (92.58%)
GM_3_IP	60,051,264	57,439,034 (95.65%)	55,753,151 (92.84%)
DM_1_IP	58,665,632	56,084,344 (95.60%)	54,441,647 (92.80%)
DM_2_IP	64,336,082	61,415,224 (95.46%)	59,505,356 (92.49%)
DM_3_IP	58,332,182	55,858,897 (95.76%)	53,975,060 (92.53%)
GM_1_input	66,147,174	63,765,876 (96.40%)	61,849,114 (93.50%)
GM_2_input	65,318,384	62,862,413 (96.24%)	60,996,560 (93.38%)
GM_3_input	58,691,190	57,200,434 (97.46%)	55,404,787 (94.40%)
DM_1_input	66,324,558	64,096,053 (96.64%)	63,950,590 (96.42%)
DM_2_input	62,785,728	60,908,435 (97.01%)	59,142,357 (94.20%)
DM_3_input	63,610,464	61,061,585 (96.61%)	59,716,094 (93.88%)

**TABLE 2:** Number of m^6^A peaks detected in bovine skeletal myoblasts and myotubes.

Sample	Total transcripts	Total m^6^A transcripts	Total m^6^A peaks	m^6^A peaks per m^6^A transcript	m^6^A peaks per transcript
GM_1	23736	6,831	11138	1.63	0.63
GM_2	23885	8,094	13596	1.68	0.77
GM_3	23287	6,042	9,246	1.53	0.53
DM_1	23815	7,275	11682	1.61	0.66
DM_2	23447	5,691	9,128	1.60	0.53
DM_3	23912	7,361	11861	1.61	0.67

To investigate the modification position of m^6^A in transcripts, we then studied the metagene profiles of the m^6^A peak in the entire transcriptome of GM and DM (Figure 2D). Three distinct m^6^A peaks were observed in the start codon, stop codon and 3′UTR (untranslated region). Simultaneously, the peak near the stop codon is significantly higher than the other two peaks. Interestingly, the m^6^A density of DM in 3′UTR is higher than GM, while the density in CDS is lower than GM. Further, to evaluate m^6^A enrichment systematically in mRNAs, we calculated the enrichment proportion of m^6^A peak in 5′UTR, CDS (coding sequence) and 3′UTR. More than 50% of m^6^A peaks are in the CDS region, nearly 40% are in the 3′UTR, and just under 10% are in the 5′UTR (Figure 2E). Moreover, the distribution pattern of mRNA m^6^A modification was highly similar in myoblasts and myotubes, and coincided with the typical m^6^A peak distribution in mammals. To further determine the distribution of m^6^A modifications in the transcriptome, we analyzed the number of m^6^A peaks contained in each gene. There was only one m^6^A peak in more than 60% of the genes, and ∼23 and 9% of the genes with 2 and 3 peaks, respectively. Only about 2–4% of the genes contain more than 4 peaks (Figure 2F). Furthermore, to investigate the abundance of m^6^A modification at the chromosome level and the difference between GM and DM, the circle diagram of m^6^A peaks was produced using OmicStudio online tools (https://www.omicstudio.cn/tool) (Figure 2G). The circular visualization of peaks on chromosomes suggested that m^6^A methylation is extensively modified in myoblasts and myotubes, but there is an inconspicuous difference in m^6^A abundance between GM and DM. Cumulative curves also showed that there was no visible difference in m6A abundance between GM and DM (Figure 2H).

### Analysis of Differentially Methylated Peaks (DMPs) Between Bovine Skeletal Myoblasts (GM) and Myotubes (DM)

**FIGURE 3:**
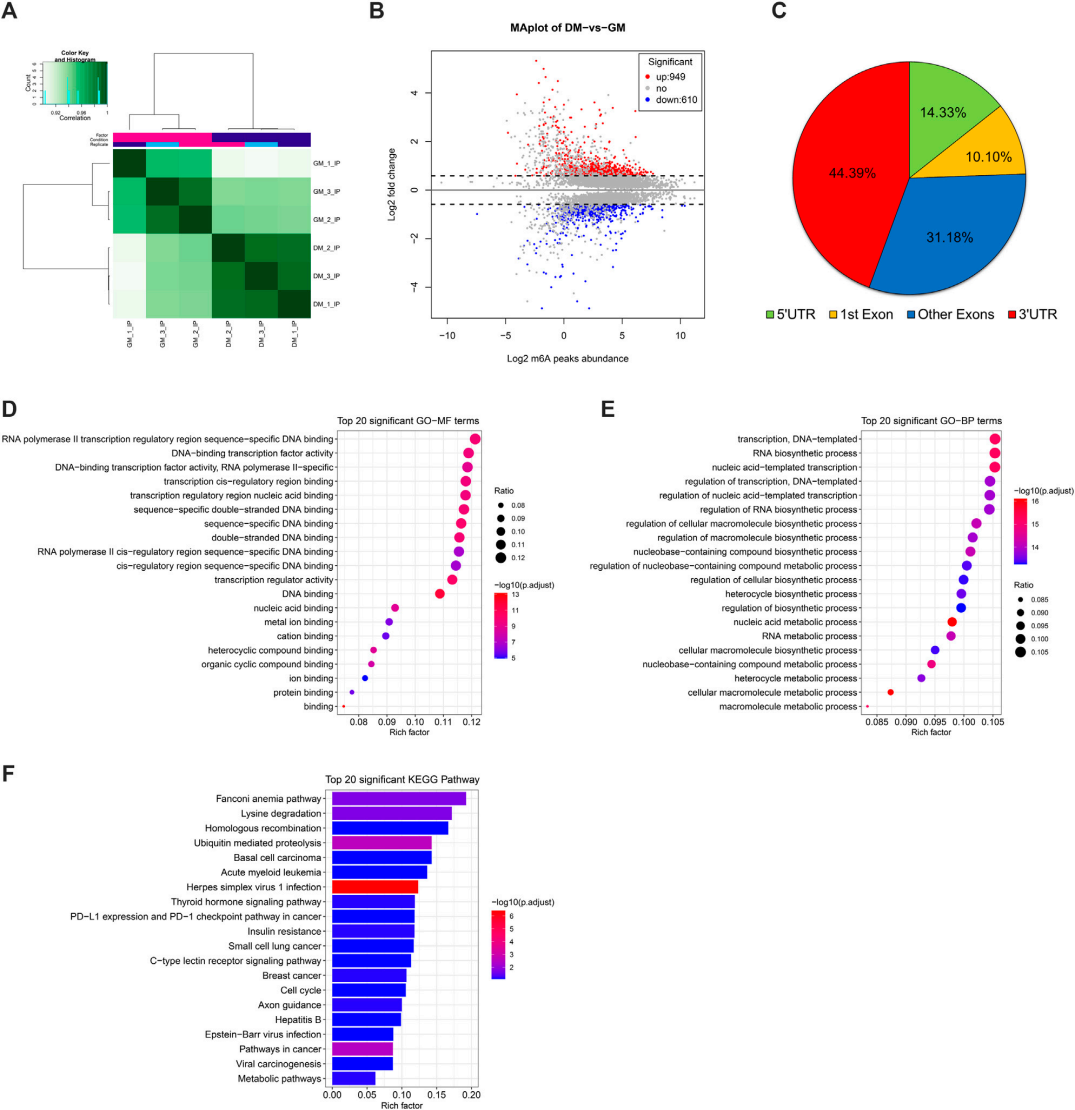
Analysis of differentially methylated peaks (DMPs) between bovine skeletal myoblasts and myotubes. **(A)** Correlation heatmap showing the correlation between GM_IP and DM_IP. **(B)** MA plot showing DMPs between DM_IP and GM_IP. **(C)** Pie chart showing the fraction of m^6^A peaks in 4 non-overlapping transcript segments (5′ UTRs, 1st exon, other exons, and 3′ UTRs). **(D)** Gene ontology (GO) enrichment analysis of molecular function for differentially methylated genes (DMGs). **(E)** GO analysis of biological process for DMGs. **(F)** KEGG analysis of DMGs.

Correlation heatmap showed that the immunoprecipitated samples were obviously divided into two treatment groups, GM_IP and DM_IP, with weak intra-group differences and significant inter-group differences visible (Figure 3A). To investigate the difference in the abundance of methylated peaks between the two groups, firstly, we identified 7,140 common peaks in both DM and GM (Supplementary Table S4). Then, 1,559 differentially methylated peaks (DMPs) were further screened, representing the annotated 1,437 differentially methylated genes (DMGs). The expression of 949 peaks augmented (correspond to 881 genes with up-regulated m^6^A abundance) and 610 peaks diminished (correspond to 571 genes with down-regulated m^6^A abundance) (Figure 3B, Supplementary Table S4). The analysis of DMPs enrichment sites showed that 44.39% of these DMPs were enriched in 3′UTR and about 41% in the exon region, while 14.33% of m^6^A modification occurs in 5′UTR (Figure 3C).

Furthermore, to analyze the potential function of m^6^A modified genes in myoblast differentiation, we performed GO [terms of molecular function (MF) and biological process (BP)] and KEGG pathway enrichment analysis for DMGs. As shown in Figures 3D,E, DMPs were mainly clustered in GO-MFs related to transcriptional regulation, including DNA binding, protein binding, transcription regulator activity and cis-regulatory region sequence (Supplementary Table S5). Likewise, they were also enriched in the GO-BPs of transcriptional regulation. Moreover, they were mainly involved in nucleic acid metabolic-related and biosynthetic-related Biological Processes. KEGG analysis indicated that DMGs enriched in disease-related signal pathways, but also participated in some signal pathways related to myogenesis, including ubiquitin mediated proteolysis, insulin resistance, cell cycle and metabolic pathways (Figure 3F, Supplementary Table S6). These results suggested that DMPs (representing DMGs) might play a role in gene transcription regulation and cell metabolism during bovine myoblast differentiation.

### Analysis of Differentially Expressed Genes (DEGs) Between Bovine Skeletal Myoblasts and Myotubes

**FIGURE 4:**
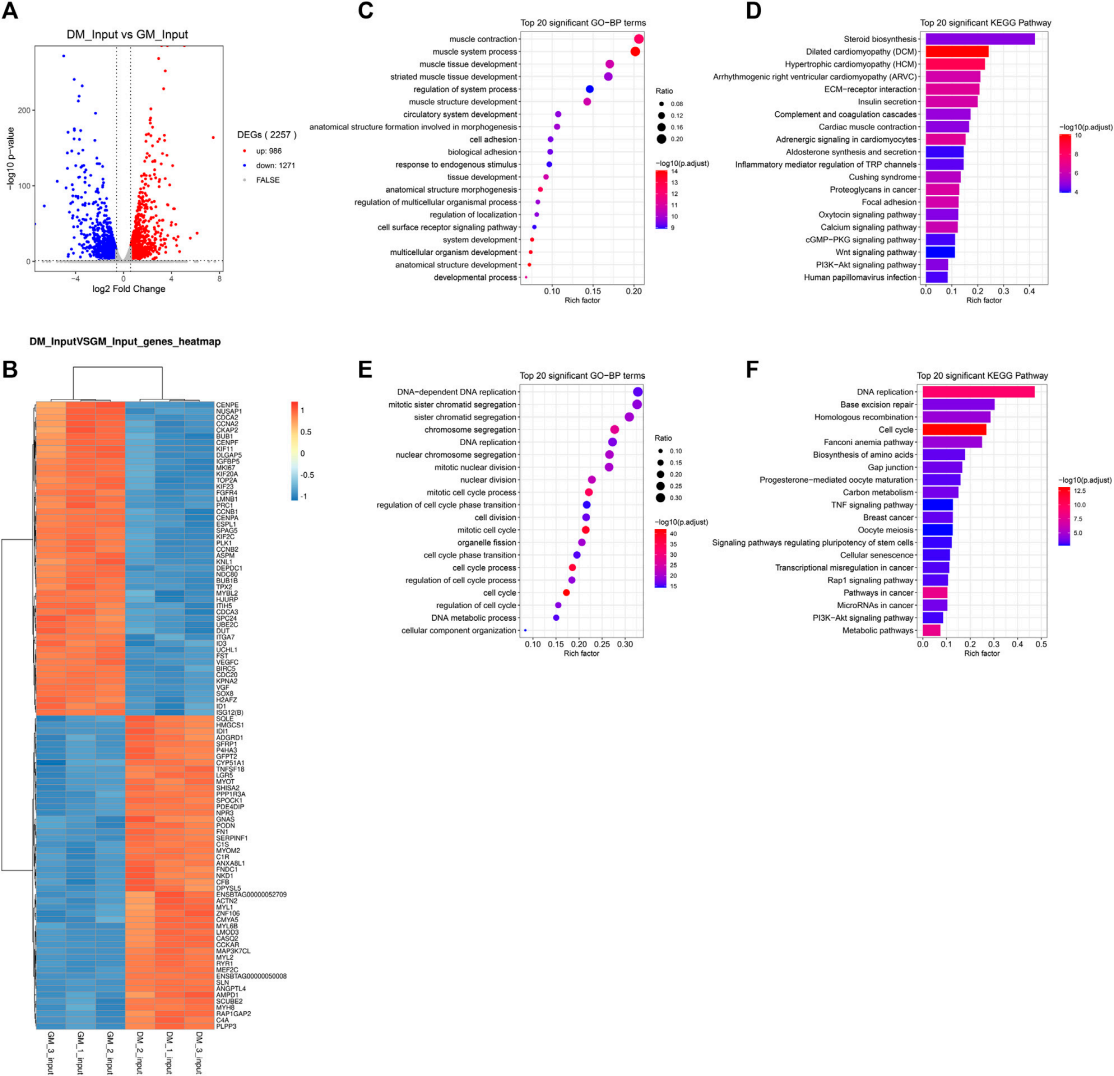
Analysis of differentially expressed genes (DEGs) between bovine skeletal myoblasts and myotubes. **(A)** The Volcano plot of DEGs. **(B)** Hierarchical clustering heatmap of DEGs. **(C)** GO analysis of biological process for up-regulated DEGs. **(D)** KEGG analysis of up-regulated DEGs. **(E)** GO analysis of biological process for down-regulated DEGs. **(F)** KEGG analysis of down-regulated DEGs.

m^6^A abundance has been reported to affect mRNA levels (Wang et al., 2014; Shen et al., 2016; Zhao et al., 2017; Zhou et al., 2019). To evaluate whether there is a potential correlation between m^6^A mRNA methylation and gene transcript levels during myoblast differentiation, RNA-seq analyses were simultaneously performed in all samples. The Volcano Plot of DEGs data were shown in Figure 4A. 2257 DEGs were identified using RNA-seq, of which 986 genes were up-regulated and 1,271 genes were down-regulated (*p* < 0.05, FC > 1.5) (Figure 4A, Supplementary Table S7). To determine the clustering pattern of genes under different experimental conditions, we took the top 50 up-regulated and top 50 down-regulated genes with the lowest *p*-value for differential gene cluster analysis (heat map) on Omicstudio tools at https://www.omicstudio.cn/tool. The hierarchical clustering of DEGs data were displayed in Figure 4B. We found that *MEF2C*, *MYL1*, *MYOM2*, *ACTN2*, *MYH8* and other genes related to myoblast differentiation and myotube formation were significantly up-regulated, while the expression of genes inhibiting muscle development was significantly decreased, including *ID1* (Jen et al., 1992), *IGFBP5* (Salih et al., 2004), *SOX8* (Schmidt et al., 2003) and *PRC1* (Bracken et al., 2006).

To further reveal the role of DEGs in myoblast differentiation, we performed GO and KEGG analysis on up-regulated and down-regulated DEGs, respectively. The results indicated, on the one hand, that the up-regulated genes are mainly enriched in biological processes closely related to myogenesis, such as muscle contract, muscle tissue development and muscle structure development (Figure 4C, Supplementary Table S8). The major signaling pathways involved include cardiomyopathy, insulin secret, cardiac muscle contract, calcium signaling pathway, Wnt signaling pathway, PI3K-Akt signaling pathway and cardiology pathway, which directly regulated muscle development (Figure 4D, Supplementary Table S9). On the other hand, the down-regulated genes were mainly clustered in cell cycle-related and cell division-related biological processes. Similarly, they were enriched in related signaling pathways of Cell cycle and Cell division, including DNA replication, cell cycle, and Rap1 signaling Pathway (Figures 4E,F, Supplementary Tables S8, 9). Meanwhile, the TNF signaling pathway and PI3K-Akt signaling pathway involved in the regulation of myogenic differentiation were enriched. These data verified that RNA-seq results were consistent with the biological process of the samples (GM and DM).

### Integrated Analysis of m^6^A-Seq and RNA-Seq Data

**FIGURE 5:**
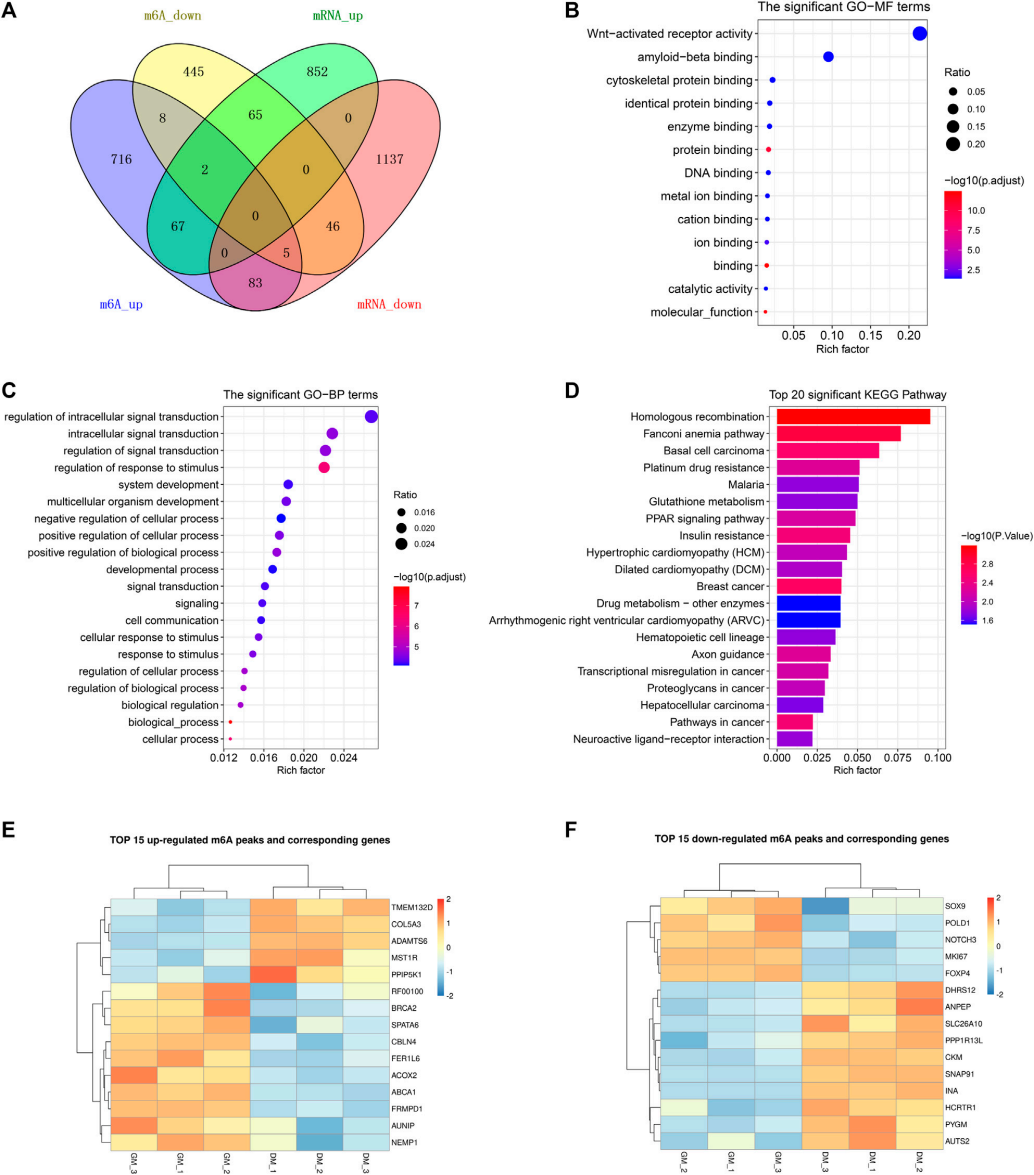
Conjoint analysis of m^6^A-seq and RNA-seq data. **(A)** Venn diagram showing the overlapping genes between DMPs and DEGs. **(B)** GO analysis of molecular function for common genes between DMGs and DEGs. **(C)** GO analysis of biological process for common genes between DMGs and DEGs. **(D)** KEGG analysis of common genes between DMGs and DEGs. **(E)** Heatmap of 15 genes mRNA expression in Table 3. **(F)** Heatmap of 15 genes mRNA expression in Table 4.

To demonstrate the potential regulation of m^6^A modification on gene expression in bovine myoblast differentiation, we analyzed genes with significant changes in both mRNA and m^6^A levels. As shown in Figure 5A, the Venn diagram of DEGs and DMPs found that a total of 268 genes had significant changes at both levels, accounting for 18.4% of 1,438 DMGs and 11.9% of 2257 DEGs. This result implied that m^6^A modification may regulate the expression of these genes during the course of myogenic differentiation. Additionally, the overlapping results of DEGs and DMPs showed that there were 67 common genes in both “m^6^A_up” and “mRNA_up” (means hyper-up), 83 common genes in both “m^6^A_up” and “mRNA_down” (means hyper-down), 65 common genes in both “m^6^A_down” and “mRNA_up” (means hypo-up), and 46 common genes in both “m^6^A_down” and “mRNA_down” (means hypo-down) (Figure 5A, Supplementary Table S10). Intriguingly, m^6^A modification abundances of 15 genes exhibited both up-regulation and down-regulation, indicating that multiple peaks with m^6^A methylation modification within these genes had different significant changes in myoblast differentiation.

Likewise, GO and KEGG analyses were performed to predict the role of the genes with significant changes in both m^6^A and mRNA levels during myoblast differentiation. GO-MF analysis showed that these genes were predominantly concentrated in binding-related pathways, including protein binding, enzyme binding, DNA binding, ion binding (Figure 5B, Supplementary Table S11). This result was similar to our results in DMPs (Figure 3D), suggesting that m^6^A modification may participate in the regulation of transcripts. Meanwhile, the results of the GO-BP analysis revealed that the genes were enriched in signal transduction, cellular process, biological process and developmental process (Figure 5C, Supplementary Table S11). KEGG analysis indicated that these genes were involved in signaling pathways related to muscle development such as cardiomyopathy and insulin resistance, besides being enriched in disease and cancer-related pathways (Figure 5D, Supplementary Table S12). As shown in Tables 3, 4, in view of the results of differential integration analysis, we listed top 15 genes with the highest up-regulated and down-regulated of m^6^A level (with significant mRNA expression level (*p* < 0.05, FC > 1.5 and FPKM >0.5) in all three biological replications of GM and DM), and Heatmaps showed differences in their mRNA expression (Figures 5E,F). Strikingly, we found that m^6^A-modified peaks of 24 of the 30 genes were enriched in 3′UTR and exons, which supported the results of the m^6^A-enriched region in this study and suggested that m^6^A may influence bovine myoblast differentiation by mediating the expression of these genes.

**TABLE 3:** Top 15 up-regulated m^6^A methylated genes between DMGs and DEGs.

Chromosome	Peak start	Peak end	Gene name	Peak annotation	log2 (fc) for m^6^A abundance	log2 (fc) for mRNA expression^a^
chr7	14587172	14587262	COL5A3	5′ UTR	2.98	1.69
chr23	25224255	25224585	RF00100	Exon	1.64	−1.54
chr8	94603892	94604732	ABCA1	Exon	1.62	−1.72
chr22	42867116	42867384	ACOX2	3′ UTR	1.51	−0.66
chr22	50369911	50370240	MST1R	3′ UTR	1.51	0.62
chr21	55277905	55278235	PPIP5K1	3′ UTR	1.46	0.59
chr13	83257828	83257947	CBLN4	5′ UTR	1.4	−1.98
chr2	127225518	127236830	AUNIP	5′ UTR	1.28	−0.70
chr8	61859199	61859528	FRMPD1	Exon	1.27	−0.67
chr14	15865318	15876727	FER1L6	Exon	1.23	−1.04
chr12	28651758	28652567	BRCA2	Exon	1.17	−0.73
chr17	47429797	47430186	TMEM132D	Exon	1.12	0.70
chr3	97926995	97942749	SPATA6	Exon	1.11	−0.60
chr5	56372089	56372269	NEMP1	3′ UTR	1.1	−0.82
chr20	14055062	14055122	ADAMTS6	5′ UTR	1.09	0.95

a FPKM >0.5 in all GM_Input and DM_Input groups.

**TABLE 4:** Top 15 down-regulated m^6^A methylated genes between DMGs and DEGs.

Chromosome	Peak start	Peak end	Gene name	Peak annotation	log2 (fc) for m^6^A abundance	log2 (fc) for mRNA expression^a^
chr7	7950177	7950476	NOTCH3	3′ UTR	−1.88	−1.31
chr2	122099045	122100956	HCRTR1	Exon	−1.51	0.93
chr12	21205865	21205984	DHRS12	3′ UTR	−1.34	0.82
chr19	58920321	58921246	SOX9	Exon	−1.25	−0.69
chr29	42985611	42985940	PYGM	3′ UTR	−1.24	0.72
chr18	53013557	53013736	PPP1R13L	Exon	−1.23	0.96
chr25	29814345	29814912	AUTS2	Exon	−1.22	0.93
chr18	52956472	52956740	CKM	3′ UTR	−1.14	2.06
chr5	55863909	55864803	SLC26A10	3′ UTR	−1.14	0.60
chr9	23517788	23518217	SNAP91	Exon	−1.11	1.24
chr21	21212454	21212842	ANPEP	5′ UTR	−1.11	0.87
chr26	47527428	47528297	MKI67	Exon	−1.07	−4.32
chr26	23899763	23900120	INA	3′ UTR	−1.07	0.74
chr23	15366336	15396428	FOXP4	5′ UTR	−1.07	−0.94
chr18	56572211	56572271	POLD1	3′ UTR	−1.06	−0.72

a FPKM >0.5 in all GM_Input and DM_Input groups.

**FIGURE 6:**
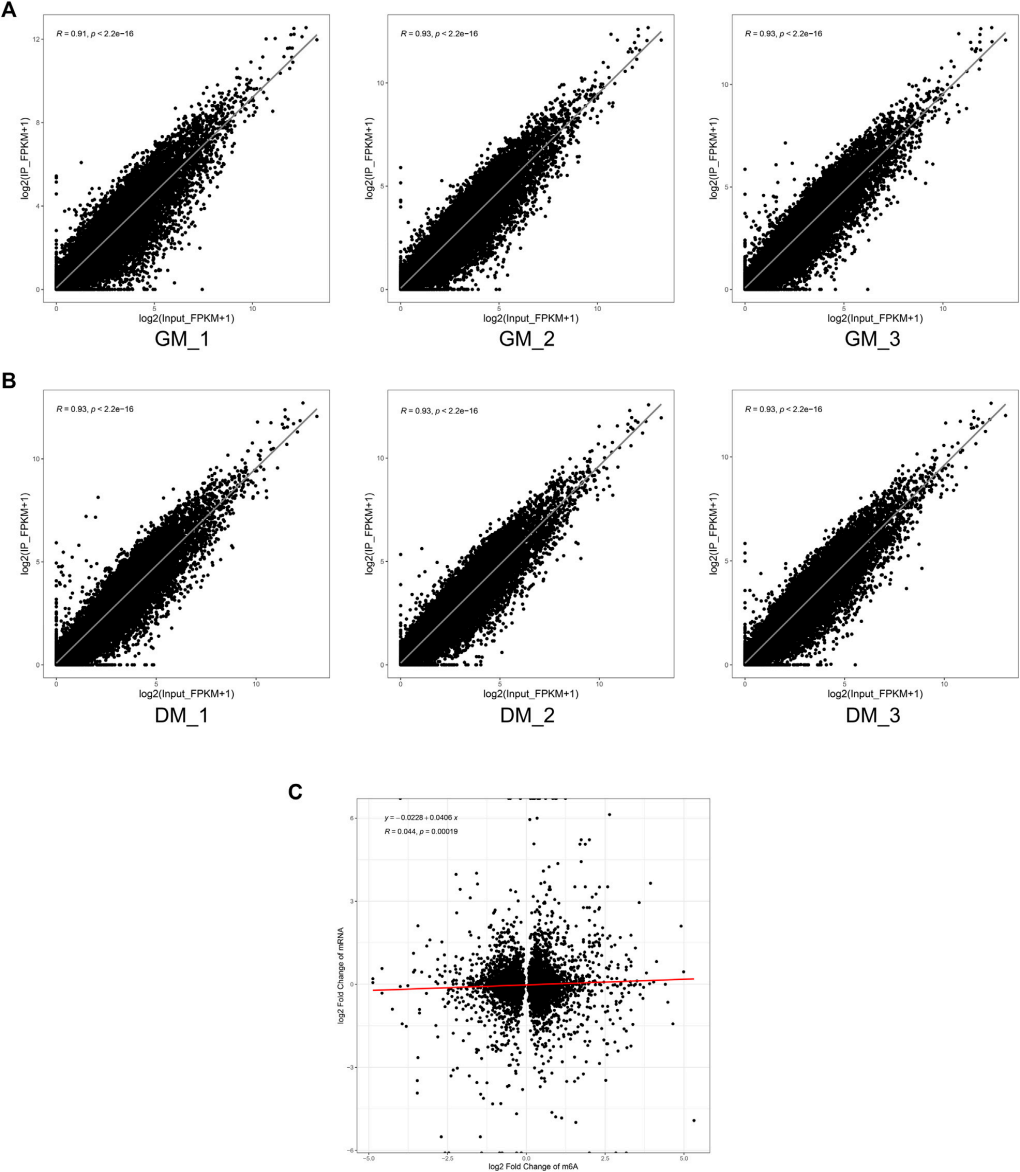
Correlation analysis of m^6^A peak enrichment and mRNA abundance in bovine myoblasts and myotubes. **(A)** Scatter plots of m^6^A peak enrichment and mRNA abundance in three biological replicates of GM showing the same tendency. **(B)** Scatter plots of m^6^A peak enrichment and mRNA abundance in three biological replicates of DM showing the same tendency. **(C)** Scatter plots showing a weak correlation between the fold changes in m^6^A methylation and mRNA expression levels for genes.

Finally, to determine whether m^6^A modification could regulate gene expression in bovine skeletal myoblasts, we analyzed the correlation between mRNA level and m^6^A peaks abundance in bovine myoblasts (GM) and myotubes (DM). Our results revealed that mRNA expression was strongly positively correlated with the abundance of m^6^A peaks in each of GM and DM (Figures 6A,B). However, there was no striking correlation between the change of m^6^A level and the differential expression of mRNA during myoblast differentiation (Figure 6C). These data suggested that the higher the expression level of the genes, the higher the abundance of m^6^A methylation modification. However, there was no significant positive or negative correlation between the changes of mRNA m^6^A methylation and differential expression during bovine skeletal myoblast differentiation.

### Validation of m^6^A and mRNA Levels of 4 Specific Myogenesis-Related Genes in Bovine Myoblast Differentiation

In the m^6^A-seq and RNA-seq analysis, we found that transcripts of several well-known myogenesis-related genes, including *MYOZ2*, *TWIST1*, *KLF5* and *MYOD1*, exhibited differential both m^6^A and mRNA levels in bovine myoblast differentiation. MYOZ2 is a muscle-specific protein in the Z-band of the sarcomere, which plays an important role in maintaining muscle fiber structure and myotube formation (Takada et al., 2001). TWIST1 is a transcription factor that inhibits skeletal myocyte differentiation (Spicer et al., 1996), while KLF5 and MYOD1 are key transcription factors that promote myogenesis (Hayashi et al., 2016).

**FIGURE 7:**
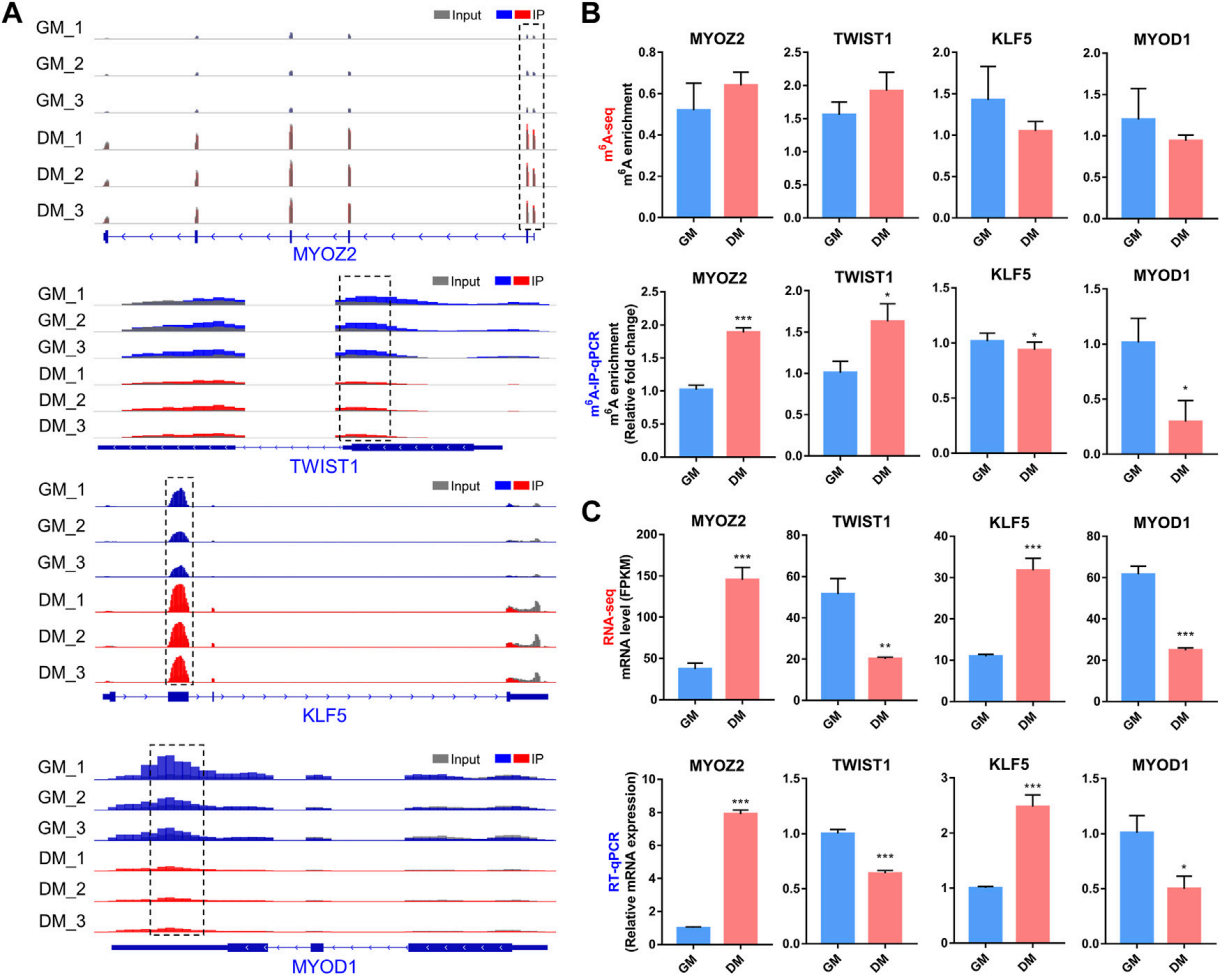
Changes in m^6^A and mRNA levels in transcripts of 4 myogenesis-related genes during bovine myoblast differentiation. **(A)** IGV tracks displaying the distribution of m^6^A peaks in MYOZ2, TWIST1, KLF5 and MYOD1 transcripts in all six groups. **(B)** Validations of the m^6^A enrichment by m^6^A-IP-qPCR. **(C)** mRNA expression levels of *MYOZ2*, *TWIST1*, *KLF5* and *MYOD1* were revealed and verified by RNA-seq and RT-qPCR, respectively. Data were presented as the means ± SD. *p < 0.05, ***p < 0.001, **p < 0.01; using Student’s *t* test.

We generated the mRNA m^6^A peaks of these four genes by using IGV software (Figure 7A). In all three biological replicates of GM and DM, m^6^A-modified peaks of *MYOZ2* and *TWIST1* were observed to be enriched near the start codon in mRNA, while m^6^A-modified peaks of KLF5 and MYOD1 were enriched within CDS and 3′UTR, respectively (Figure 7A). We performed m^6^A-IP-qPCR to verify the results of m^6^A-seq and indicated that the mRNAs of *MYOZ2* and *TWIST1* displayed higher levels of m^6^A enrichment in DM compared with GM, and the mRNAs of *KLF5* and *MYOD1* exhibited lower levels of m^6^A enrichment in DM (Figure 7B). Additionally, RNA-seq and RT-qPCR revealed and verified the transcript levels of *MYOZ2* and *KLF5* were enhanced significantly in DM compared with GM, and *TWIST1* and *MYOD1* were diminished significantly in DM (Figure 7C). These results confirmed the accuracy of the m^6^A-seq and RNA-seq data. What’s more, these data provided possible mechanisms for m^6^A methylation to regulate myoblast differentiation, and these four genes may also be considered as downstream candidate targets for m^6^A modification during myoblast differentiation.

## Discussion

The discovery of the first m^6^A demethylase FTO (Jia et al., 2011) and the development of m^6^A-specific high-throughput sequencing technology (Dominissini et al., 2012; Meyer et al., 2012) provided theoretical basis and technical support for the study of m^6^A in plants and animals, respectively. Related studies in farm animals have also been uncovered in recent years. However, as far as we know, the research on m^6^A modification in cattle has not been reported, and the enrichment pattern and potential role of m^6^A methylation in beef cattle muscle development were still unclear. In the present study, we identified transcriptome-wide *N* ^6^-methyladenosine profiling of bovine skeletal myoblast differentiation by performing m^6^A-seq and RNA-seq. Bioinformatics analysis suggested the potential role of m^6^A modification in regulating myoblast differentiation. Further experiments verified the accuracy of the sequencing results, and screened some myogenesis-related genes with obvious m^6^A methylation modification.

First of all, we verified that the bovine skeletal muscle myoblasts isolated in this study could carry out normal myoblast differentiation by observing myotube formation and using RT-qPCR. The results of RNA-seq later indicated that DEGs were mainly involved in the process of muscle growth and development, which also proved the reliability of sequencing. Our results showed that m^6^A methylation occurred in 24.3–33.9% transcripts during bovine skeletal myoblast differentiation, and these transcripts with m^6^A modification contained more than one m^6^A peak on average. The results suggest that m^6^A is widely modified in bovine myoblasts and may play an important role in skeletal myogenesis. Similar to other mammals, m^6^A peaks in myoblasts and myotubes were also mainly enriched in motif RRACH. This result was consistent with the findings in pigs (Tao et al., 2017; Jiang et al., 2019), chickens (Cheng et al., 2021) and geese (Xu et al., 2021), but different from the results in plants (Wei et al., 2018; Zhou et al., 2019), suggesting that m^6^A modification in mammals may be similar to birds, but different from plants. Exploring the sequence and location of motifs could provide a reference for subsequent molecular mechanism studies. The motifs are catalyzed by m^6^A methylases and recognized and bound by m^6^A reader proteins. Many studies have shown that m^6^A modification is reduced after specific mutation of m^6^A motif (Guo et al., 2020; Ye et al., 2020), which would provide support for subsequent investigations into the potential role of m^6^A modification. In our study, four genes were selected for m^6^A-IP-qPCR validation, and the primers used were also designed in view of m^6^A motif positions.

The distribution of m^6^A peaks in bovine myoblasts is similar to that in humans, mice and pigs, mainly abundant near the stop codon, CDS and 3′UTR (Dominissini et al., 2012; Tao et al., 2017; Gheller et al., 2020; Zhang et al., 2020), but is inconsistent with the result that m^6^A peaks were mainly distributed near the start codon in chicken fat and goose muscle (Cheng et al., 2021; Xu et al., 2021). The results demonstrated a difference in the distribution of m^6^A methylation in mammals and birds. Previous studies have shown that m^6^A modification in different regions of mRNA may have different mechanisms. m^6^A within the 5′UTR regulated cap-independent translation in stress response, and CDS and 3′UTR m^6^A was recognized by YTHDF2 or IGF2BPs to degrade and stabilize the target mRNAs, respectively (Shi et al., 2019). Besides, YTHDF1 was more inclined to bind to the m^6^A site of 3′UTR to promote translation (Shi et al., 2019). Our results revealed that among the TOP15 genes with the highest up-regulated or down-regulated m^6^A level, a total of 24 genes had m^6^A peaks distribution in the 3′UTR and CDS regions, and m^6^A modification occurred in 5′UTR of the only six genes. As shown in Table 4, *CKM* mRNA was upregulated in the bovine myoblast differentiation, while the mRNA m^6^A abundance was diminished. It is reasonable to speculate that YTHDF1 or IGF2BPs may recognize the m^6^A site in 3′UTR of *CKM* mRNA and promote its mRNA translation or stabilization. CKM is a terminal differentiation gene for myogenesis (Millay et al., 2013), suggesting that m^6^A may influence myoblast differentiation by mediating the m^6^A level of CKM. Subsequent experiments will be conducted to verify the molecular mechanism.

GO analysis revealed that DMGs were commonly enriched in DNA binding, protein binding and transcription-related terms of Molecular Function, and Biological Process enrichment analysis demonstrated that DMGs were mainly associated with transcriptional regulation, nucleic acid metabolism and RNA metabolism. These results are consistent with previous results of m^6^A modification in C2C12 myoblast differentiation (Gheller et al., 2020) and porcine skeletal muscle development (Tao et al., 2017; Zhang et al., 2020), suggesting that m^6^A modification may be involved in gene transcription regulation and RNA metabolism during myoblast differentiation. KEGG analysis in this study showed that DMGs were involved in insulin resistance and metabolic pathways, among which STAT3, JAK2, IGF2, CKM, PIK3CA and other genes that regulate skeletal muscle development were scanned, implying a new potential mechanism of myogenesis-related genes regulating the bovine myoblast differentiation. The previous study has found that the m^6^A level of both C2C12 and primary mouse myoblasts on the third day of differentiation was significantly lower than that in the proliferation phase (Gheller et al., 2020). In our study, there was no significant difference in the m^6^A abundance of GM and DM, which may be related to the interference of some genes with low abundance on the sequencing results. Subsequent experiments, such as LC-MS/MS or dot blot, are required to determine the changes of m^6^A level during bovine myoblast differentiation.

The associated analyses of m^6^A-seq and RNA-seq revealed that there was a strong positive correlation between mRNA m^6^A abundance and expression level in each GM and DM group. The higher the gene expression level, the higher the m^6^A abundance. The result is contrary to the idea that m^6^A and mRNA expression in chicken adipose tissue was negatively correlated (Cheng et al., 2021). We speculate that it may be attributed to differences in species and cell types. Notably, integrated analysis for differential expression showed that there was no marked correlation between fold changes of mRNA expression and fold change of m^6^A level during bovine myoblast differentiation. Inconsistent with our findings, changes in m^6^A methylation were negatively correlated with gene expression during goose embryonic muscle development (Xu et al., 2021). We analyzed the m^6^A-seq data of chicken fat deposition and found that there was a positive correlation between differential m^6^A abundance and differential gene expression level (Cheng et al., 2021). In addition, the two modules with positive or negative correlation with body traits were screened by WGCNA during porcine embryonic muscle development, and the combined analysis displayed that there was either a significant positive or negative correlation between m^6^A methylation changes and gene expression multiples in the individual module (Zhang et al., 2020). These results suggest that the association between mRNA m^6^A methylation abundance and gene expression level may be conservative in species, but also related to the differences between tissue level *in vivo* and cellular level *in vitro*.

Finally, the m^6^A level and mRNA relative expression of four well-known myogenesis-related genes were verified by m^6^A-IP-qPCR and RT-qPCR. The differentially expressed m^6^A peaks in *TWIST1* mRNA were enriched near 5 ′UTR, and the m^6^A level was up-regulated while the mRNA level was down-regulated, suggesting that m^6^A may inhibit *TWIST1* mRNA expression *via* promoting m^6^A methylation of *TWIST1* mRNA to promote myoblast differentiation due to TWIST1 inhibited skeletal muscle differentiation (Spicer et al., 1996). Previous studies showed that no m^6^A modification was found in *Myod1* mRNA in mESC (Batista et al., 2014), whereas m^6^A methylation of *Myod1* in C2C12 was significantly enriched in 5′UTR, and siMETTL3 led to a restraint of myoblast differentiation by reducing *MYOD1* mRNA expression (Kudou et al., 2017). In contrast, our study found that m^6^A modification of *MYOD1* mRNA was enriched in 3′UTR and its mRNA expression was decreased, which may have different or similar regulatory mechanisms in bovine myoblast differentiation. In view of previous reports and our data, it is reasonable to speculate that the role of m^6^A modification in skeletal muscle differentiation may not be a pure promoting or inhibiting mechanism, but rather may be complex. Our ongoing research project will be to investigate the molecular mechanism of m^6^A modification regulating skeletal myogenesis *in vitro* using bovine myoblasts as a model.

## Conclusion

In conclusion, we first revealed the mRNA m^6^A modification map in bovine skeletal myoblasts and myotubes. We found that m^6^A methylation may regulate myogenesis *via* mediating the gene expression. Further, four candidate target genes were identified and screened, including MYOZ2, TWIST1, KLF5 and MYOD1. These comprehensive analyses open a new perspective for the genetic improvement and molecular breeding of beef cattle, and provide a theoretical basis for studying the functional and molecular mechanism of m^6^A methylation in regulating skeletal muscle development and myogenesis.

## Data Availability

The datasets supporting our findings are included in the article and Supplementary Material. The m^6^A-seq and RNA-seq data generated by this study have been deposited to GEO database under accession number GEO: GSE173477.
