# Supplementary material for: Transcriptome-wide N 6-Methyladenosine Methylome Profiling Reveals m6A Regulation of Skeletal Myoblast Differentiation in Cattle (Bos taurus)
Source: Front Cell Dev Biol. 2021 Dec 6;9:785380. doi: 10.3389/fcell.2021.785380 (PMC8685427; doi:10.3389/fcell.2021.785380)
Supplement: Supplementary file 2 [file Table1.DOCX]

Supplementary Table 1. Primers used in this study

| Primer name | Sequence (5’-3’) |
| --- | --- |
| Primers for RT-qPCR | |
| qGAPDH-F | AGTTCAACGGCACAGTCAAGG |
| qGAPDH-R | ACCACATACTCAGCACCAGCA |
| qMYOD1-F | AACCCCAACCCGATTTACC |
| qMYOD1-R | CACAACAGTTCCTTCGCCTCT |
| qMYOG-F | GGCGTGTAAGGTGTGTAAG |
| qMYOG-R | CTTCTTGAGTCTGCGCTTCT |
| qMYH3-F | TGAACGCCCTCTCCAAATCC |
| qMYH3-R | AATGAAGTGCTGTCTCGGCA |
| qMYMK-F | TCGGCCATCCTCATCATTG |
| qMYMK-R | CGTACGTGTAGTCCCAGTCCTC |
| qMRF4-F | GTGATAACTGCCAAGGAAGGAG |
| qMRF4-R | CGAGGAAATGCTGTCCACGA |
| qCKM-F | CAACATGAAGGAGGTTTTCCG |
| qCKM-R | GGTTAGATGGGCAGGTGAGC |
| qMYOZ2-F | AACAGAGTTGCCACCCCATT |
| qMYOZ2-R | CCATCCCTTCGGAGTCCTATT |
| qTWIST1-F | CTACCAGGTCCTACAGAGCGA |
| qTWIST1-R | CTCCATCCTCCAGACCGAGAA |
| qKLF5-F | GAGCTGGTCCAGACAAGATGT |
| qKLF5-R | TCAGGTGAGTGATGTCAGGGA |
| Primers for m^6^A-IP-qPCR | |
| MYOZ2-m^6^A-F | TCACCGGAACGAATTCCACT |
| MYOZ2-m^6^A-R | GCCCATCCTTCAGAGAGTCG |
| TWIST1-m^6^A-F | CTACCAGGTCCTACAGAGCGA |
| TWIST1-m^6^A-R | CTCCATCCTCCAGACCGAGAA |
| KLF5-m^6^A-F | GCTCCTGAATACACCGGATCT |
| KLF5-m^6^A-R | GAATTGCTTCATGGCGGTCG |
| MYOD1-m^6^A-F | GCCCCTCGGGCTGTATTTAT |
| MYOD1-m^6^A-R | TTCGAACACCTGAGCGAGC |
